# 0681. Effects of veno-venous co2 removal therapy on pulmonary circulation in an ARDS model

**DOI:** 10.1186/2197-425X-2-S1-P45

**Published:** 2014-09-26

**Authors:** P Morimont, T Desaive, J Guiot, V Tchana-Sato, N Janssen, A Cagnina, S Habran, S Kosta, D Hella, F Blaffart, P Kolh, J-O Defraigne, B Lambermont

**Affiliations:** University Hospital of Liege, Liege, Belgium; University of Liege, GIGA-Research, Cardiovascular Sciences, Liege, Belgium

## Introduction

Acute respiratory distress syndrome (ARDS) is responsible for injuries to the alveolar epithelium and microvascular endothelium resulting in hypoxemia, decreased pulmonary compliance and increased pulmonary vascular resistance. Beneficial effects resulting from protective lung ventilation are counterbalanced by deleterious hemodynamic effects. Indeed, hypercapnia resulting from ventilation at lower tidal volume enhances pulmonary hypertension and is associated with right ventricular failure in ARDS[1].

## Objectives

The aim of our study was to determine if low flow CO2 removal therapy used at early stage of ARDS could have beneficial hemodynamic effects on pulmonary circulation.

## Methods

This study was performed on an experimental model of ARDS obtained in 6 pigs. After sedation, analgesia and endotracheal intubation via a cervical tracheostomy, the pigs were connected to a volume-cycled ventilator. A micromanometer-tipped catheter was inserted into the main pulmonary artery and into the left atrium. Systemic arterial blood pressure (AP) was monitored via a micromanometer-tipped catheter inserted into the abdominal aorta through the left femoral artery. A conductance micromanometer-tipped catheter was inserted into the right ventricle. ARDS was obtained by repeated bronchoalveolar lavage (0.9% saline solution). Protective ventilation at lower tidal volume was then achieved. 12 Fr aspiration and 10 Fr injection cannulas were inserted into the inferior and the superior vena cava, respectively. These cannulas were connected to a pump-driven extracorporeal membrane oxygenator (PALP, MAQUET, Germany) in order to achieve CO2 removal therapy.

## Results

ARDS induced severe hypercapnic acidosis (PaCO2 = 82.5 ± 10.8 vs. 46.3 ± 10.7 mm Hg and pH = 7.14 ± 0.07 vs. 7.46 ± 0.07, p < 0.01). Systolic pulmonary artery pressure (PAPs) significantly increased from 27.4 ± 4.3 to 43.0 mm Hg, p < 0.01. After the PALP was started, acidosis was corrected (pH = 7.39 ± 0.08) and normocarbia was maintained (PaCO2 = 41.9 ± 12.6 mm Hg, p < 0.01) despite protective ventilation (tidal volume = 6 vs. 10 mL/kg). At the same time, PAPs significantly decreased to 30.8 ± 5.0 mm Hg, p < 0.01. Cardiac output, left atrial pressure as well as AP did not significantly change. Mean PALP blood flow was 645 mL/min.

## Conclusions

Veno-venous removal therapy enabled protective ventilation while maintaining normocarbia during ARDS. CO2 removal decreased pulmonary hypertension and improved right ventricular function. This technique may be an effective lung- and right ventricular- protective adjunct to mechanical ventilation.
